# Comment on Iasella et al. Close Follow-Up of Patients with Neurofibromatosis Type 1 Reduces the Incidence of Malignant Peripheral Nerve Sheath Tumour. *Cancers* 2025, *17*, 1306

**DOI:** 10.3390/cancers17203355

**Published:** 2025-10-17

**Authors:** Walter Taal, Rianne Oostenbrink, Martinus P. G. Broen, Anja M. M. Gijtenbeek, Marie-Claire Y. de Wit

**Affiliations:** 1 Department of Neurology, Erasmus MC Cancer Institute, 3015 GD Rotterdam, The Netherlands; 2 Department of Pediatrics, Erasmus MC Sophia Children’s Hospital, 3015 GD Rotterdam, The Netherlands; r.oostenbrink@erasmusmc.nl; 3 Department of Neurology, Maastricht UMC+, 6229 HX Maastricht, The Netherlands; martijn.broen@mumc.nl; 4 Department of Neurology, Radboud UMC, 6525 GA Nijmegen, The Netherlands; anja.gijtenbeek@radboudumc.nl; 5 Department of Pediatric Neurology, Erasmus MC Sophia Children’s Hospital, 3015 GD Rotterdam, The Netherlands; m.c.y.dewit@erasmusmc.nl

We read with great interest the recent study by Iasella et al., titled “*Close Follow-Up of Patients with Neurofibromatosis Type 1 Reduces the Incidence of Malignant Peripheral Nerve Sheath Tumour*” [1]. The authors address a highly relevant clinical question regarding active surveillance with whole-body MRI (WBMRI) in adults with Neurofibromatosis Type 1 (NF1) and its proposed effect on reducing the incidence of malignant peripheral nerve sheath tumors (MPNSTs). While the data presented are compelling, we believe several critical methodological limitations warrant a more cautious interpretation of the findings.

The study’s retrospective design and reliance on a historical control group limit its ability to establish causality. The comparison is made against a Finnish cohort from 1987 to 2012, a period that largely predates the routine use of MRI, FDG-PET, and molecular diagnostics. This raises concerns about diagnostic sensitivity and case ascertainment—particularly given that the reported NF1 prevalence in that cohort (1:3850) is substantially lower than the widely accepted figure of approximately 1:2000. This discrepancy suggests that milder cases may have been underrepresented, potentially biasing the historical incidence of MPNST upwards.

We also note the absence of detailed reporting on potential harms associated with the surveillance strategy. Specifically, it would be informative to know how many incidental findings were identified, how many led to further investigations, and what the associated burden was in terms of patient anxiety, time, and healthcare utilization. Additionally, clarification is needed regarding the clinical justification for the removal of 28 benign tumors, including data on surgical outcomes such as complications or functional impairment. Furthermore, some patients in the reported cohort presented with pain or other alarming symptoms—cases in which imaging and timely intervention might have occurred within a surveillance protocol that includes regular clinical visits to monitor symptoms without standard WBMRI for all patients.

Lastly, we note the lack of discussion around quality of life—a key consideration when evaluating any screening or surveillance intervention, particularly in a chronic condition such as NF1. Incidental findings on WBMRI that lead to follow-up scans, biopsies, or surgery result in costs for both patients and society, as well as stress and risk of complications. These risks must be weighed against the potential benefit of earlier malignancy detection.

In light of these limitations, we advise caution in interpreting the study’s conclusions. While the findings are intriguing and hypothesis-generating, we believe that broader implementation of WBMRI in NF1 surveillance should await confirmation from prospective, controlled studies that comprehensively assess both benefits and harms.
